# Expression of the zinc-finger transcription factor Osterix (SP7) in invasive breast cancer and its prognostic significance

**DOI:** 10.1007/s13402-025-01062-9

**Published:** 2025-04-07

**Authors:** Behnaz Saidy, Laura Gull, Andrew G. Hacker, Emad A. Rakha, Andrew R. Green, Ian O. Ellis, Stewart G. Martin, Sarah J. Storr

**Affiliations:** 1 Nottingham Breast Cancer Research Centre, School of Medicine, Biodiscovery Institute, University of Nottingham, University Park, Nottingham, NG7 2RD UK; 2 Milton Keynes University Hospital, Standing Way, Eaglestone, Milton Keynes, MK6 5LD UK; 3 Division of Cancer and Stem Cells, School of Medicine, Biodiscovery Institute, Nottingham Breast Cancer Research Centre, University of Nottingham, University Park, Nottingham, NG7 2RD UK

**Keywords:** Breast cancer, Osterix, SP7

## Abstract

**Introduction:**

Osterix, encoded by SP7, is a transcription factor crucial in osteoblast differentiation and bone formation. While initially characterised in bone development, emerging evidence suggests its involvement in cancer, particularly breast cancer metastasis to bone.

**Methods:**

Osterix protein expression was evaluated in 1340 early-stage invasive breast tumours by immunohistochemistry. Cytoplasmic and nuclear expression levels were assessed and associations with clinicopathological variables and patient survival determined. Additionally, *SP7* mRNA expression was examined in the METABRIC cohort of patients. Gene set enrichment analysis (GSEA) was performed to explore the role of osterix in the hallmarks of cancer genesets.

**Results:**

Results revealed significant associations between reduced nuclear osterix protein expression and adverse clinicopathological features, including larger tumour size, higher grade, and poor Nottingham Prognostic Index. Low nuclear osterix protein expression was also linked to shorter breast cancer-specific survival and distant metastasis free survival, particularly in patients with HER2 positive tumours. No associations were found between *SP7* mRNA expression and clinicopathological variables or survival outcomes. GSEA identified enrichment of genes involved in KRAS signaling in tumours with high *SP7* expression.

**Conclusion:**

These data suggest that reduced nuclear expression of osterix is associated with poor clinical outcome of breast cancer patients and may be of clinical relevance.

## Introduction

Osterix, also known as Sp7 and encoded by *SP7*, is a member of the Sp subgroup of Krüppel-like family of zinc finger transcription factors (XKLF) that feature a three zinc-finger structure, and is crucial in osteoblast differentiation and intricate process of bone formation (reviewed in [1]). Abnormalities associated with osterix can lead to the development of conditions such as recessive types of osteogenesis imperfecta. Osterix was initially described in 2002 with initial studies in postnatal mice revealing the indispensable nature of osterix expression for bone formation during growth and into adulthood [2, 3].

The regulatory pathways governing osterix expression encompass both Runt-related transcription factor (Runx)2-dependent routes, typically activated by bone morphogenic proteins (BMPs), and Runx2-independent pathways, stimulated by BMPs, transforming growth factor (TGF)-β, and fibroblast growth factor (FGF) (reviewed in [1]). The involvement of osterix in cancer-associated osteolytic lesions was first noted in tandem with the recognition of p53 as a negative regulator of osterix [4]. Subsequent findings demonstrated that osterix can inhibit Wnt and β-catenin signalling [5], and acts as BMP-2 induced transcription factor [6]. Activation of c-Src also increases transcriptional activity of osterix [7].

There is a growing body of evidence that indicates that osterix may also be involved in breast cancer metastasis, particularly in the process of metastatic colonisation in bone. Initial findings revealed that osterix expression was associated with the increased expression of genes linked to metastasis, including vascular endothelial growth factor (VEGF), matrix-metalloproteinase (MMP)-9 and β-catenin [8]. Osterix has been shown to activate VEGF promoter activity, and directly bind to the VEGF promoter in osteoblasts [9]. There have been subsequent in vitro and in vivo reports of osterix involvement in various steps of breast cancer metastasis [10], and small scale studies determining osterix expression in breast tumours yielding inconsistent prognostic value.

In a study of 154 tumours from breast cancer patients, osterix expression was associated with an unfavourable survival outcome and the presence of lymph node metastasis [10]. This research also showed that overexpression of osterix in MDA-MB-231 breast cancer cells promoted invasiveness, partly through upregulation of MMP9 expression [10]. In a later study examining 112 breast tumours, osterix expression did not show significant associations with any available clinicopathological criteria; however, consistent with earlier reports, osterix overexpression in MDA-MB-231 was associated with increased cellular migration [11]. This study also utilised proteomics to identify 19 differentially expressed proteins in cells with altered osterix expression, including S100A4, with evidence suggesting that osterix induced migration was partially mediated by S100A4; this study also demonstrated a correlation between expression of osterix and S100A4 in breast tumours [11].

Bone is a frequent destination of breast cancer metastasis, and the establishment of a pre-metastatic niche, orchestrated by alterations initiated by the primary cancer at a distant metastatic site to promote metastasis, is associated with the expression of osterix. Osterix, along with Runx2, are expressed within the microenvironment of microscopic breast cancer lesions; suggesting that the niche exhibits features of osteogenesis [12]. Data has shown that extracellular vesicles derived from breast cancer cells inhibit the expression of osteoblast marker genes, including osterix [13]. Additionally, osterix has also been identified as one of the differentially expressed genes upregulated in decalcified bone metastases when compared to the matched primary breast cancer [14].

Other than the documented effects on metastasis, osterix overexpression has been shown to increase the sensitivity of breast cancer cells to doxorubicin or paclitaxel, partly through the upregulation of GALNT14 [15]. Studies have also revealed a decrease in osterix expression in bone samples from rats treated with lapatinib, a dual ErbB1/ErbB2 inhibitor, and paclitaxel, resulting in bone loss and bone marrow adiposity [16].

The objective of this study was to evaluate osterix protein and *SP7* mRNA expression in a large and comprehensively annotated group of breast cancer patients, to determine associations with patient survival and clinicopathological parameters.

## Methods

### Western blotting

Western blotting was used to confirm antibody specificity and suitability for use in immunohistochemical studies. Gel electrophoresis was performed using the Invitrogen Bolt mini system, with 4–12% Bis–Tris plus gels. Breast cancer cell lysates were prepared in Bolt LDS sample buffer and Bolt sample reducing buffer and denatured by incubation at 100 °C for five minutes. Transfer to nitrocellulose (Whatman, GE Healthcare) was achieved using Bolt transfer buffer with 10% methanol. Following blocking in 3% non-fat milk for one hour nitrocellulose was incubated with anti-beta-actin (Abcam AB8226) and anti-osterix antibody (Abcam, ab22552) antibodies overnight at 4 °C. Secondary antibodies, donkey anti-rabbit immunoglobulin (Li-Cor 926-32213, 1:10000) and donkey anti-mouse immunoglobulin (Li-Cor 926-68072 1:10000) were incubated for one hour prior to visualisation on an Odyssey FC Imager (Li-Cor) using Image Studio software (V4.1).

### Patient cohort and immunohistochemistry

This study involved 1340 tumours from early stage, primary operable, invasive breast cancer patients treated at Nottingham University Hospitals between 1998 and 2006. All patients underwent breast conserving surgery or mastectomy, decided by disease characteristics or patient choice, followed by radiotherapy if indicated. Nottingham Prognostic Index (NPI), oestrogen receptor (ER) and menopausal status determined if patients received systemic adjuvant treatment. Patients with an NPI score less than 3.4 did not receive adjuvant treatment, and patients with an NPI score of 3.4 and above were candidates for cyclophosphamide, methotrexate and 5-fluorouracil (CMF) combination chemotherapy if they were ER-negative or pre-menopausal; and hormonal therapy if they were ER-positive. No patients received trastuzumab. Ethical approval was issued by Nottingham Research Ethics Committee 2, under the title of ‘development of a molecular genetic classification of breast cancer’ (C202313). Breast cancer-specific survival was calculated as the time interval between primary surgery and death resultant from breast cancer. Metastasis free survival was defined as the date of surgery to metastasis. Immunohistochemistry was performed on tissue microarrays that were comprised of single 0.6 mm cores taken from representative tumour areas selected by a specialist breast cancer histopathologist from haematoxylin and eosin-stained sections. This study is reported according to REMARK criteria [17].

Tissue microarray sections, each 4 μm thick, were deparaffinised and subsequently rehydrated in a sequential immersion process in xylene, ethanol and water. Antigen retrieval was conducted in 0.01 mol L^− 1^ sodium citrate buffer (pH 6.0), with tissue heated in a microwave for 10 min at 750 W, followed by 10 min at 450 W. Staining was performed using a Novolink Polymer Detection kit (Leica) adhering to the manufacturer’s instructions and described previously [18, 19]. Anti-osterix antibody (Abcam, ab22552, 1:500) was incubated on tissue for one hour at room temperature. After staining, tissue was dehydrated in ethanol and fixed in xylene before mounting using DPX. Stained slides were scanned using a Nanozoomer Digital Pathology Scanner (Hamamatsu Photonics) at 200x magnification.

Osterix staining in the cytoplasm was assessed using a semi-quantitative immunohistochemical H score; where the percentage area of tumour cells with a staining intensity of 0 to 3, representing none, weak, intermediate and strong intensity staining. The final H-score for each tumour was assessed multiplying the intensity score by the percentage of positive cells. Osterix nuclear staining was scored as the percentage of tumour cells demonstrating any level of staining. More than 30% of cores for each tissue microarray were double assessed, with both scorers blinded to clinical outcome and each other’s scores. Independent scorers have a single measure intraclass correlation coefficient of > 0.700 indicating good concordance between assessors.

### METABRIC cohort

Details of the METABRIC dataset (*n* = 1980) have been documented elsewhere [20]. Cohort specimens were obtained from five facilities in the UK and Canada spanning the years 1977 to 2005, with consent obtained from the respective institutional review boards as detailed in the original publication [20]. Breast cancer-specific survival was computed as the duration between primary surgery and death attributed to breast cancer. The majority of patients who were ER negative and had positive lymph nodes underwent adjuvant chemotherapy, while those who were ER positive and/or with positive lymph nodes did not. Trastuzumab was not administered to patients who had HER2 overexpressing tumours. Data analysis was performed on mRNA expression z-scores relative to all samples (log microarray) downloaded from http://www.cbioportal.org [21–23].

### Statistical analyses

Statistical analyses were performed using IBM SPSS software (version 28) with cases stratified according to breast cancer specific survival using X-tile software for both protein and mRNA expression [24]. *P* values less than 0.05 were considered statistically significant. The association of osterix expression with clinicopathological variables was determined using the Pearson χ ^2^ test of association, to examine whether there was a linear trend between the levels of ordinal and categorical variables, a linear by linear χ ^2^ test was performed. The Kaplan-Meier method was used to produce survival curves using log-rank test to determine significance. Multivariate survival analysis was performed using Cox’s proportional hazard method. Gene set enrichment analysis (GSEA) was performed using GSEA software, with samples divided by categorised *SP7* expression levels (https://www.gsea-msigdb.org/gsea/index.jsp) and gene enrichment determined in the curated hallmarks of cancer gene sets (h.all.v2023.2.Hs.symbols.gmt) [25–28]. Broad Institute Morpheus software was used to visualize data (https://software.broadinstitute.org/morpheus).

## Results

### Osterix protein expression in invasive breast cancer

**Fig. 1:**
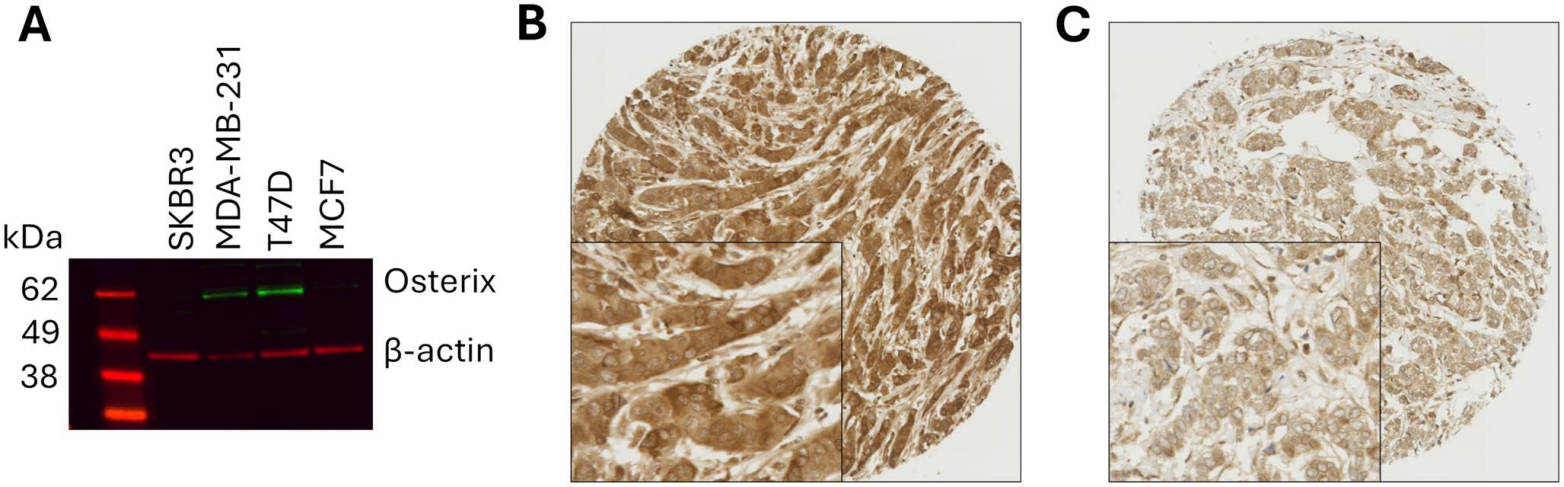
(**A**) Western blot assessment of osterix expression in breast cancer cell lines. Representative photomicrographs of high osterix immunohistochemical staining (**B**), and low staining (**C**), where photomicrographs are shown at 10× magnification with 20× magnification inset box

Antibody specificity was confirmed using Western blotting prior to staining patient tissues (Fig. 1A). 1340 breast tumours were examined for both cytoplasmic and nuclear osterix expression; representative tissue staining is shown in Fig. 1B and C. Expression of osterix within the cytoplasm was significantly correlated with osterix expression in the nucleus (*P* < 0.001; R^2^ = 0.435).

The median H-score for cytoplasmic expression of osterix was 140 (ranging between 50 and 240), and the median percentage score for nuclear osterix expression was 30 (ranging between 0 and 90). X-tile was used to generate cut points, with a cut point of 185 used for cytoplasmic osterix expression and a cut point of 25 used for nuclear osterix expression. Nuclear and cytoplasmic osterix expression were significantly correlated with one another (*P* < 0.001, R^2^ = 0.435) but not with strong biological relevance.

### Osterix protein expression and association with clinicopathological parameters

Associations between cytoplasmic and nuclear expression of osterix protein expression and clinicopathological variables were assessed. Low levels of nuclear expression of osterix were found to be significantly associated with clinicopathological parameters characteristic of aggressive behavior using Pearson χ^2^ test of association, including larger tumour size (χ^2^ = 4.479, d.f.=1, *P* = 0.034), ER negative tumours (χ^2^ = 8.165, d.f.=1, *P* = 0.004), progesterone receptor (PR) negative tumours (χ^2^ = 7.876, d.f.=1, *P* = 0.005), triple receptor negative tumours (χ^2^ = 5.177, d.f.=1, *P* = 0.023), positive vascular invasion (χ^2^ = 6.506, d.f.=1, *P* = 0.011) and histological type (χ^2^ = 16.738, d.f.=4, *P* = 0.002), at the time of presentation; data is shown in Table 1. Linear by linear χ ^2^ tests were performed to identify linear trends; low levels of nuclear expression of osterix had a significant trend with increasing tumour grade (χ^2^ = 12.969, d.f.=2, *P* < 0.001), increasing pleomorphism variation score (χ^2^ = 7.120, d.f.=2, *P* = 0.008), and worse NPI prognostic group (χ^2^ = 10.831, d.f.=2, *P* < 0.001) at the time of presentation; data is shown in Table 1.

**Table 1:** Associations between the cytoplasmic and nuclear expression of Osterix determined using immunohistochemistry with clinicopathological variables. The *P* values are resultant from pearson Χ^2^ test of association, or linear by linear Χ^2^ test if an * is present, and significant values (*P* < 0.05) are highlighted in bold. ER is oestrogen receptor and PgR is progesterone receptor

	Cytoplasmic osterix expression			Nuclear osterix expression		
	Low	High	*P* value	Low	High	*P* value
**Age**						
< 50 years	322 (78.3%)	90 (21.8%)	0.241	199 (48.3%)	213 (51.7%)	0.891
≥ 50 years	751 (80.9%)	177 (19.0%)		444 (47.9%)	483 (52.1%)	
**Tumour size**						
< 2.0 cm	634 (77.9%)	180 (22.1)	**0.013**	372 (45.7%)	442 (54.3%)	**0.034**
≥ 2.0 cm	439 (83.5%)	87 (16.5%)		271 (51.6%)	254 (48.4%)	
**Grade**						
1	132 (68.0%)	62 (32.0%)	< 0.001*	71 (38.6%)	123 (63.4%)	< 0.001*
2	422 (79.2%)	111 (20.8%)		253 (47.6%)	279 (52.4%)	
3	519 (84.7%)	94 (15.3%)		319 (52.0%)	294 (48.0%)	
**Pleomorphism**						
1	12 (60.0%)	8 (40.0%)	0.053*	4 (20.0%)	16 (80.0%)	0.008*
2	288 (78.5%)	79 (21.5%)		163 (44.5%)	203 (55.5%)	
3	773 (81.1%)	180 (18.9)		476 (49.9%)	477 (50.1%)	
**Mitosis**						
1	485 (76.6%)	148 (23.4%)	0.011*	281 (44.5%)	351 (55.5%)	0.053*
2	228 (84.1%)	43 (15.9%)		144 (53.1%)	127 (46.9%)	
3	360 (82.6%)	76 (17.4%)		218 (50.0%)	218 (50.0%)	
**Vascular invasion**						
Negative	760 (79.3%)	198 (20.7%)	0.281	439 (45.8%)	519 (54.2%)	**0.011**
Positive	313 (81.9%)	69 (18.1%)		204 (53.5%)	177 (46.5%)	
**Stage**						
1	657 (79.8%)	166 (20.2%)	0.300*	383 (46.5%)	440 (53.5%)	0.120*
2	297 (78.4%)	82 (21.6%)		188 (49.0%)	191 (54.4%)	
3	118 (86.1%)	19 (13.9%)		72 (52.9%)	64 (47.1%)	
**NPI**						
Good	342 (77.0%)	102 (23.0%)	0.011*	185 (41.7%)	259 (58.3%)	< 0.001*
Medium	542 (80.3%)	133 (19.7%)		340 (50.4%)	335 (49.6%)	
Poor	188 (85.5%)	32 (14.5%)		118 (53.9%)	101 (46.1%)	
**ER status**						
Negative	209 (77.7%)	60 (22.3%)	0.278	150 (55.8%)	119 (44.2%)	**0.004**
Positive	863 (80.7%)	207 (19.3%)		492 (46.0%)	577 (54.0%)	
**PR status**						
Negative	461 (82.5%)	98 (17.5%)	0.06	293 (52.4%)	266 (47.6%)	**0.005**
Positive	863 (78.3%)	207 (21.7%)		345 (44.6%)	428 (55.4%)	
**HER2 status**						
Negative	930 (80.0)	232 (20.0%)	0.953	547 (47.1%)	614 (52.9%)	0.104
Positive	142 (80.2%)	35 (19.8%)		95 (53.7%)	82 (46.3%)	
**Triple negative**						
Negative	909 (80.0%)	227 (20.0%)	0.978	529 (46.6%)	606 (53.4%)	**0.023**
Positive	153 (80.1%)	38 (19.9%)		106 (55.5%)	85 (44.5%)	
**Lymph node status**						
Negative	657 (79.8%)	166 (20.2%)	0.79	383 (46.5%)	440 (53.5%)	0.16
Positive	415 (80.4%)	101 (19.0%)		260 (50.5%)	255 (49.5%)	
**Ki67 index Groups**						
Low	406 (77.6%)	117 (22.4%)	0.231	233 (44.6%)	290 (55.4%)	0.419
High	389 (80.7%)	93 (19.3%)		227 (47.1%)	255 (52.9%)	
**Histological subtype**						
None Special Type (NST)	735 (83.0%)	151 (17.0%)	**< 0.001**	454 (51.3%)	431 (48.7%)	**0.002**
Invasive Lobular Carcinoma	87 (80.6%)	21 (19.4%)		46 (42.6%)	62 (57.4%)	
Metaplastic Carcinoma	3 (100.0%)	0 (0.0%)		3 (100.0%)	0 (0.0%)	
Pure Special Type	33 (58.9%)	23 (14.1%)		19 (33.9%)	37 (66.1%)	
Mixed NST	215 (74.9%)	72 (25.1%)		121 (42.2%)	166 (57.8%)	

Similarly, low levels of cytoplasmic osterix expression were significantly associated with larger tumour size (χ^2^ = 6.220, d.f.=1, *P* = 0.013) and histological type (χ^2^ = 25.863, d.f.=4, *P* < 0.001) at the time of presentation using Pearson χ ^2^ test of association; data is shown in Table 1. Linear by linear χ ^2^ tests were performed to identify linear trends; low levels of cytoplasmic expression of osterix had a significant trend with increasing tumour grade (χ^2^ = 12.969, d.f.=2, *P* < 0.001), increasing mitotic count score (χ^2^ = 6.458, d.f.=2, *P* = 0.011), and worse NPI prognostic group (χ^2^ = 6.412, d.f.=2, *P* = 0.011) ) at the time of presentation; data is shown in Table 1.

### Osterix protein expression and patient outcome

**Fig. 2:**
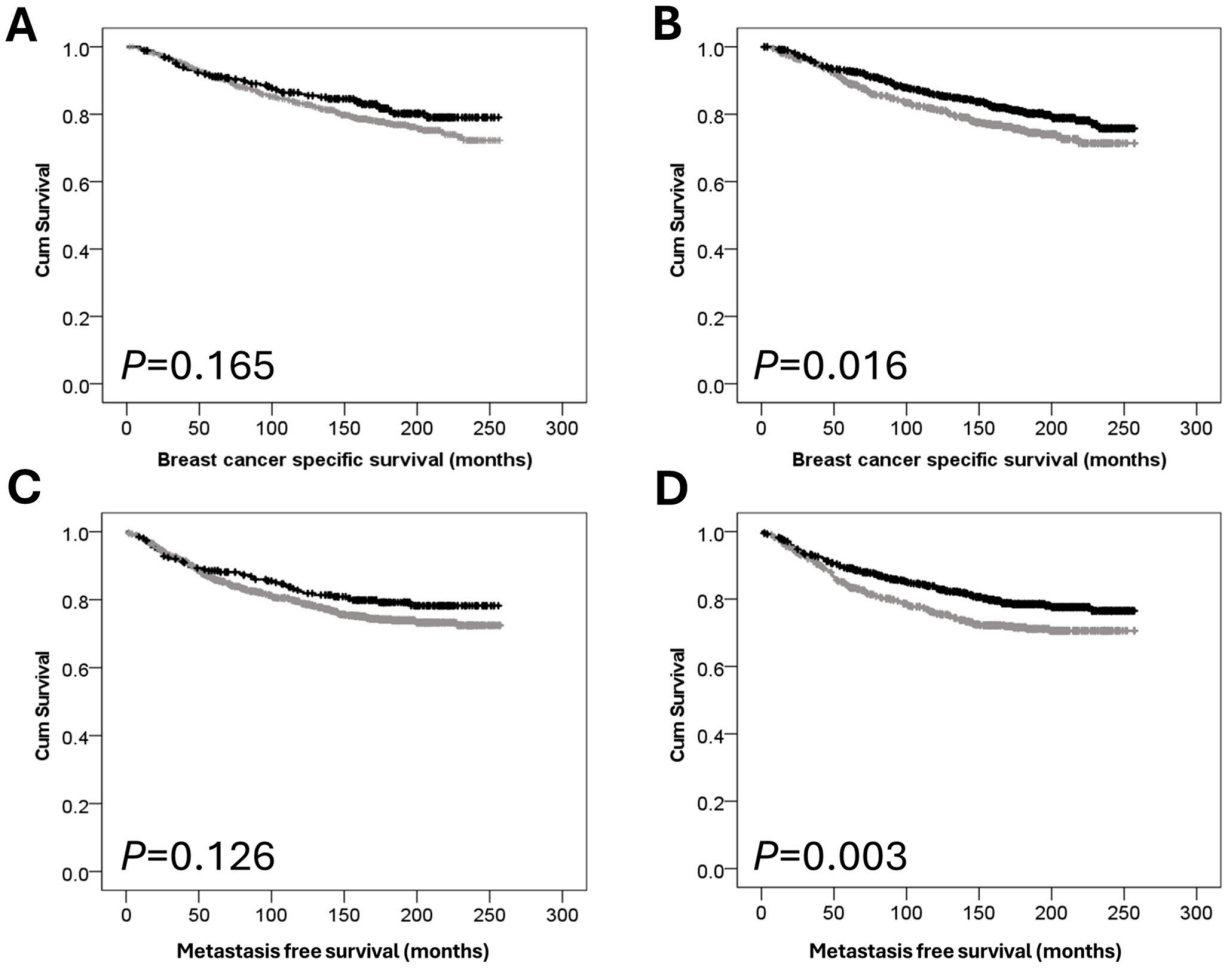
Kaplan–Meier analysis of breast cancer specific survival showing the impact of low (grey line) vs. high (black line) protein expression: (**A**) osterix cytoplasmic expression; (**B**) osterix nuclear expression of low (grey line) and high (black line) protein expression: (**C**) osterix cytoplasmic expression; (**D**) osterix nuclear expression

Kaplan-Meier analysis was used to assess the impact of osterix protein expression on breast cancer-specific survival. Low nuclear osterix expression was significantly associated with adverse breast cancer specific survival (*P* = 0.016) (Fig. 2B) and shorter distant metastasis free survival (*P* = 0.003) (Fig. 2D). Cytoplasmic osterix expression was not associated with disease specific patient survival (*P* = 0.165) (Fig. 2A) or distant metastasis free survival (*P* = 0.126) (Fig. 2C).

Multivariate survival analysis was performed using Cox’s proportional hazard method, and included tumour size, tumour grade, and stage, low nuclear osterix expression was independently associated with distant metastasis free survival (hazard ratio (HR) = 0.777, 95% confidence interval (CI) = 0.614–0.982, *P* = 0.034), but not disease specific survival (HR = 0.826, 95% CI = 0.644–1.058, *P* = 0.130).

**Fig. 3:**
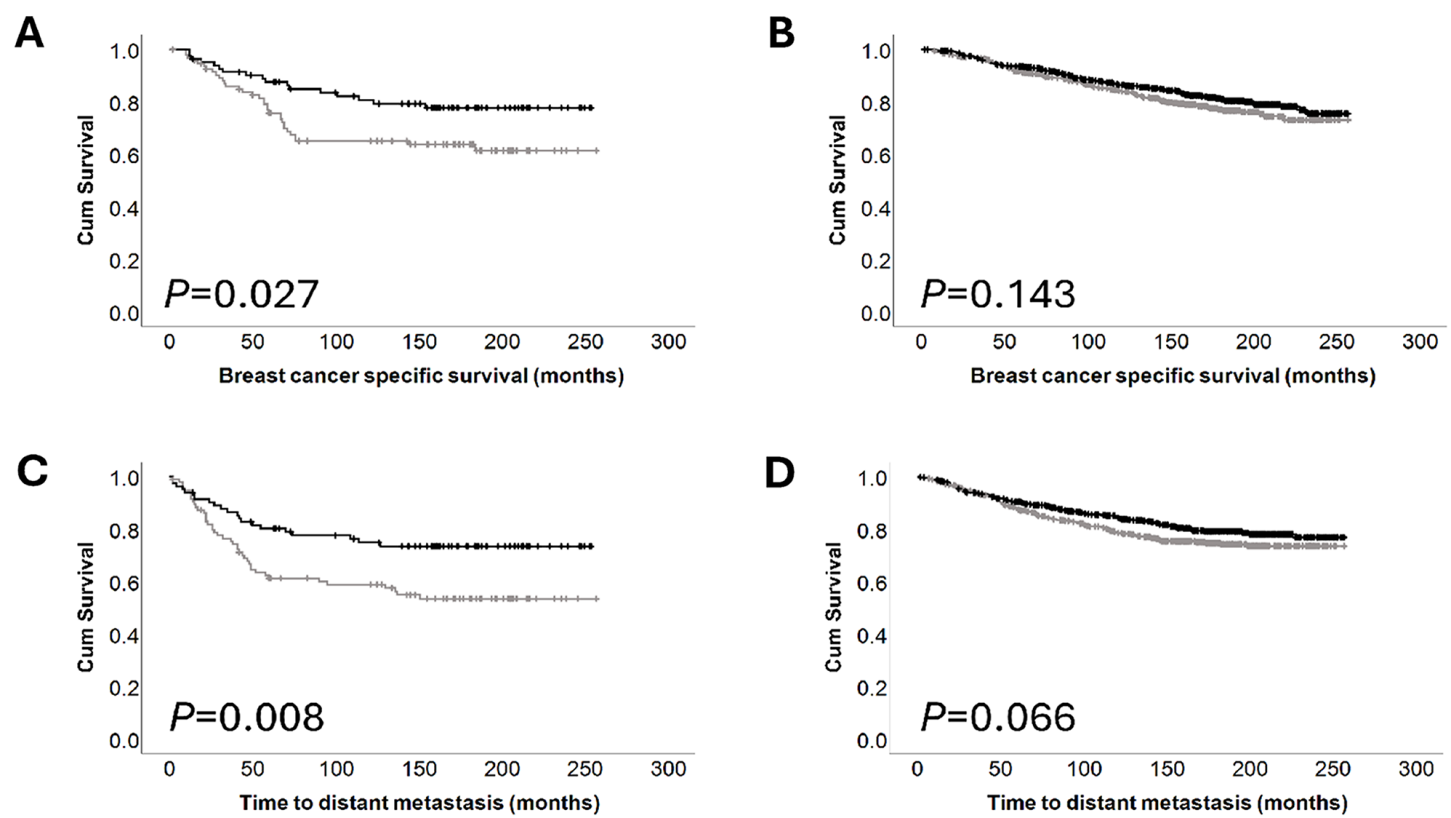
Kaplan–Meier analysis of breast cancer specific survival showing the impact of low (grey line) vs. high (black line) nuclear osterix expression: (**A**) HER2 positive tumours; (**B**) HER2 negative tumours; (**C**) HER2 positive tumours; (**D**) HER2 negative tumours

The effect of osterix expression on clinical outcome in patient subgroups was explored. Low nuclear expression of osterix was significantly associated with both disease free survival and metastasis free survival in patients with HER2 positive tumours (*P* = 0.027 and *P* = 0.008 respectively), but not in patients with HER2 negative tumours (Fig. 3).

### SP7 mRNA expression in breast cancer

**Fig. 4:**
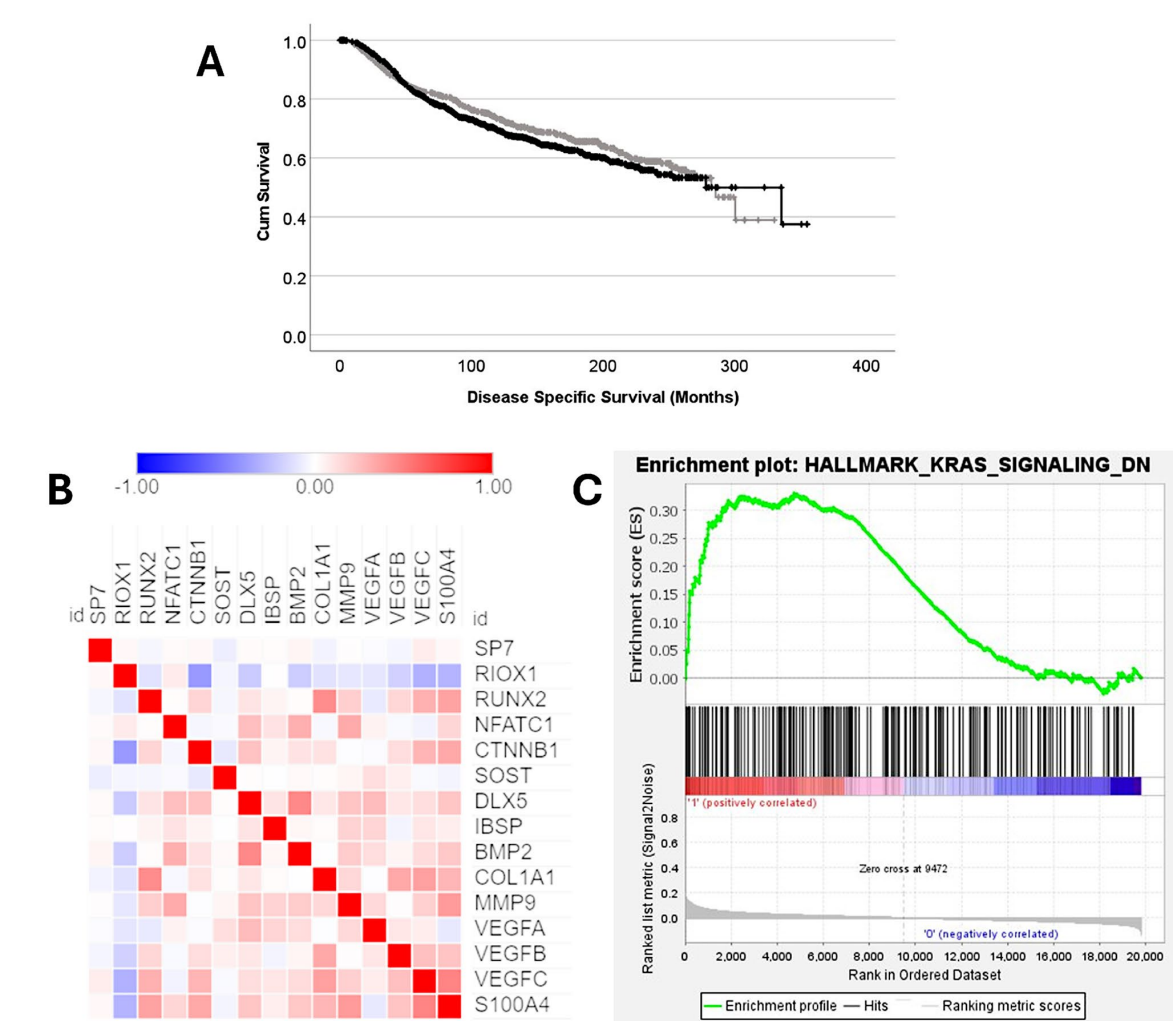
(**A**) Kaplan–Meier analysis of breast cancer specific survival showing the impact of low (grey line) vs. high (black line) *SP7* mRNA expression; (**B**) correlation matrix demonstrating Pearson correlation between *SP7* mRNA and network partners in the METABRIC cohort; (**C**) gene enrichment plot of hallmark geneset significantly enriched in high *SP7* mRNA group

*SP7* mRNA expression was analysed in the METABRIC patient cohort, which has limited overlap of patient tumours assessed within the tissue microarray. Data analysis was performed on mRNA expression z-scores relative to all samples with a median expression of -0.485 ranging from − 3.20 to 14.54. X-tile was used to generate a cut point of -0.20 to dichotemise data into low and high expression, with 847 cases having low *SP7* mRNA expression and 1134 cases having high *SP7* expression. Expression of *SP7* was not associated with survival in the total patient cohort (Fig. 4A). Associations between *SP7* expression and clinicopathological variables were assessed, but no significant associations were observed (Table 2).

**Table 2:** Associations between mRNA expression of *SP7* and clinicopathological variables in the METABRIC patient cohort. The *P* values are resultant from pearson Χ^2^ test of association and significant values (*P* < 0.05) are highlighted in bold. ER is oestrogen receptor and PgR is progesterone receptor

	SP7 mRNA expression		
	Low	High	*P* value
**Tumour size**			
≤ 2 cm	379 (19.4%)	473 (24.2%)	0.187
> 2 cm	457 (23.4%)	644 (33.0%)	
**ER status**			
Negative	189 (9.5%)	285 (14.4%)	0.150
Positive	657 (33.2%)	849 (42.9%)	
**PR status**			
Negative	396 (20.0%)	544 (27.5%)	0.608
Positive	450 (22.7%)	590 (29.8%)	
**HER2 status**			
Negative	747 (37.5%)	986 (49.8%)	0.369
Positive	99 (5.0%)	148 (7.5%)	
**PAM 50 + Claudin low subtype**			
Claudin low	89 (4.5%)	129 (6.5%)	0.406
Luminal A	296 (15.0%)	404 (20.5%)	
Luminal B	222 (11.2%)	253 (12.8%)	
HER2	88 (4.5%)	136 (6.9%)	
Basal	89 (4.5%)	120 (6.1%)	
Normal	59 (3.0%)	89(4.5%)	
**Lymph node status**			
No nodes positive	443 (23.3%)	550 (28.9%)	0.185
Any nodes positive	379 (19.9%)	532 (27.9%)	
**Nottingham Prognostic Index**			
Good	305 (15.4%)	375 (18.9%)	0.384
Moderate	458 (23.1%)	643 (32.5%)	
Poor	83 (4.2%)	116 (5.9%)	

### SP7 mRNA expression with known network partners

Gene expression data from network partners identified in String (v12.0), or through publication (VEGF, MMP9, S100A4) were assessed for correlation with *SP7* (Fig. 4B). *SP7* mRNA expression was not significantly associated with expression of *RIOX1*, *RUNX2*, *NFATC1*, *CTNNB1*, *DLX5*, *IBSP*, *BMP2*, *COL1A1*, *MMP9*, *VEGFA*, *VEGFB*, and *S100A4*; but expression was significantly, but weakly negatively correlated with *SOST* (R^2^=-0.067, *P* = 0.003), and weakly positively correlated with *VEGFC* (R^2^ = 0.072, *P* = 0.001) expression.

### Gene enrichment analysis

GSEA was used to explore the METABRIC microarray data for enrichment of genes in the curated hallmarks of cancer gene sets in high and low *SP7* expressing tumours. Normalised enrichment scores (NES) with a false discovery rate of less than 1% identified 1/50 enriched gene sets in tumours with high expression of *SP7*, but none in tumours with low expression of *SP7*. The gene set enriched in high *SP7* expressing tumours was the geneset that represents genes down-regulated by K-Ras activation (HALLMARK_KRAS_SIGNALLING_DN), with an NES of 1.58 (*P* = 0.0058) (Fig. 4C).

## Discussion

This study has demonstrated that low osterix protein expression in the nucleus, but not cytoplasm, was associated with adverse disease specific survival and time to distant metastasis in a large, well annotated cohort of breast cancer patients (*P* = 0.016 and *P* = 0.003 respectively). There was no association between cytoplasmic osterix protein expression and disease specific breast cancer survival and time to distant metastasis, nor *SP7* mRNA expression and disease specific survival. The finding that protein expression, and not mRNA expression, is associated with survival is not unusual, with post-translational regulation, cellular location and function, all reasons why this observation can be made. The research presented in this study demonstrates the largest investigation of osterix expression in primary breast tumours to date. Although this study investigates links between osterix expression and clinical outcome, it does not describe a mechanistic role for osterix within tumours, which warrants further investigation.

Whilst research is starting to demonstrate that osterix may play a role in cancer, particularly in the process of bone metastasis, there have been limited studies that have determined the expression of osterix in primary breast tumours. Previous research that investigated osterix expression in 154 tumours from breast cancer patients demonstrated that high expression was associated with an unfavourable survival outcome and the presence of lymph node metastasis [10]; however, a later study examining 112 breast tumours did not show any significant associations with any available clinicopathological criteria. The findings within this study, are different to those reported by Yao et al. (2019) [10], who reported high osterix expression was significantly associated with adverse patient survival (*P* = 0.048); however, it is important to note that there are a number of differences between these studies, including over 70% of tumours being larger than 2 cm at time of presentation in the cohort of 154 patients, and a focus on overall survival rather than disease specific survival in the same study [10]. Interestingly, research investigating gene expression in matched primary breast cancers and decalcified bone metastases has identified osterix as one of the differentially expressed genes upregulated in bone metastasis; indicating a significant expression change occurs during the process of metastasis to the bone [14]. This study also identifies that osterix expression levels are associated with survival of patients with HER2 positive tumours rather than HER2 negative tumours; whilst it is unclear why this is the case, osterix can be activated by ERK signalling, which is activated downstream of HER2.

This study has demonstrated that it is nuclear osterix expression and not cytoplasmic osterix expression that is associated with unfavourable clinical outcomes. Expression of osterix within the nucleus has been demonstrated in osteogenic cells, whereas cytosolic localisation is predominant within non-osteogenic cells; following osterix activation, its translocation to the nucleus results in activation of downstream genes and interaction with other transcription factors [29]. In this study, although statistically significant, nuclear and cytoplasmic osterix expression were not very strongly correlated (R^2^ = 0.435); this finding indicates that there is a functional switch to nuclear localisation. Interestingly, although low cytoplasmic and nuclear expression of osterix was associated with more aggressive clinicopathological variables, only low nuclear osterix expression was associated with adverse survival; this further supports the functional importance of osterix within the nucleus. The antibody used within this study is a polyclonal antibody that has been utilised in a number of previous publications [30–32].

In addition to assessing the impact of osterix expression on patient survival, we also sought to determine associations between osterix expression and clinicopathological criteria. In this study, low osterix protein expression, with cytoplasmic or nuclear localisation, was associated with larger tumour size, higher tumour grade, mitosis, pleomorphism, and poor NPI values at the time of presentation. No associations were seen between *SP7* mRNA expression and clinicopathological variables in the METABRIC cohort. As indicated earlier, nuclear osterix expression was more strongly associated with clinical outcome of patients with HER2 positive tumours.

This study has determined that mRNA expression of *SP7* was significantly correlated with networks partners sclerostin (SOST) and VEGFC in the METABRIC cohort; however, although significant, were of limited biological relevance with R [2] values of -0.067 and 0.0072 respectively. In the METABRIC patient cohort, *SP7* expression is weakly negatively correlated with *SOST*; this was unexpected as SOST has been shown to be a direct target of osterix in osteocytes [2]. High sclerostin expression has been shown to be associated with breast cancer bone metastasis; perhaps most interestingly in light of findings presented within this study, sclerostin has been shown to interact with STAT3 to enhance TGF-β/K-Ras signalling [33]. GSEA identified that high expression of *SP7* mRNA is linked with genes down-regulated by K-Ras activation in the hallmarks of cancer gene sets. K-Ras functionally activates many cellular pathways, including ERK and mTOR amongst others, and ERK1/2 is known to activate osterix transcriptional activity [34]. During mechanical stress-induced signalling in osteoblastic cells, osterix expression is increased, in addition to Runx2 activation dependent upon Ras and ERK1/2 activation [35]. K-Ras signalling has been shown to act as a positive regulator of osterix expressing early osteoprogenitor cells [36].

This study has demonstrated that low nuclear expression of osterix is associated with adverse disease-specific patient survival and time to distant metastasis, whilst cytoplasmic osterix expression and *SP7* mRNA expression were not associated with patient outcome. These findings warrant further investigation in larger patient cohorts and indicate that osterix may be of clinical relevance in breast cancer.

## Data Availability

Data analysis was performed on mRNA expression z-scores relative to all samples (log microarray) downloaded from http://www.cbioportal.org.
